# Peer support for patients with type 2 diabetes in rural communities of China: protocol for a cluster randomized controlled trial

**DOI:** 10.1186/1471-2458-14-747

**Published:** 2014-07-23

**Authors:** Bo Xie, Xiu-li Ye, Zi-lin Sun, Min Jia, Hui Jin, Chang-ping Ju, Li Yao, Carvalho Husni Da Costa De Vladmir, Yanxiaoxiao Yang

**Affiliations:** Department of Endocrinology, Zhongda Hospital, Institute of Diabetes, Medical School, Southeast University, Nanjing, 210009 China; Department of Epidemiology and Health Statistics, Southeast University, Nanjing, 210009 China

**Keywords:** Peer support, Diabetes, Self-management, Rural health

## Abstract

**Background:**

The prevalence of diabetes has been growing rapidly in developing countries. This causes devastating economic burdens and increases demands on the health care system. Therefore, there is an urgent need to find a cost-effective and multi-faceted approach for diabetes care. Peer support models provide a potentially low-cost, flexible means which complements the current existing health care services. In this way, trained peer leaders can become qualified extensions to a formal healthcare system, capable of assisting education delivery and bolstering the efforts of professional staff. As such, creating a cultural specific peer support program and determining whether it is acceptable and cost-effective in rural communities of China is crucial. This study aims to implement and evaluate biophysical and psychosocial outcomes of peer support program for people with type 2 diabetes in rural communities, and to explore the program’s feasibility and sustainability in China.

**Methods/Design:**

This study is a cluster randomised controlled trial. All consenting patients will be randomised by community staff members to receive either peer support or the control care. The data collection and analysis including social demographics, health status, psychosocial status, economic status and biomedical measures will be collected at baseline, 6 months, and 12 months. The primary indicator measured is the change in HbA1c, whereas secondary indicators include biophysical, psychosocial functioning and other lifestyle factors. Finally, economic evaluations will determine whether the program is cost effective.

**Discussion:**

This protocol is a cluster randomized, controlled trial of group-based peer support for people with type 2 diabetes in the community settings of rural China. Results from this trial may provide evidence to the effectiveness of peer support; furthermore, they will provide valuable information concerning the acceptability and feasibility of a new approach to improve diabetes self-management among resource-constrained settings.

**Trial registration:**

ClinicalTrials.gov Identifier: NCT02119572, April 18, 2014.

## Background

Diabetes is a growing health problem worldwide. It is estimated that 382 million people live with diabetes around the world, and by 2035, that number will surge to 592 million [1]. Diabetes mellitus imposes a huge economic burden on national health care systems globally. The global health expenditures for diabetes in 2030 is forecasted to increase 30% to 34% from 2010. Developing countries will experience a twenty-year growth rate of 67% while the developed countries will have a rate of 27% [2]. A systematic review showed that diabetes affects 6.8%, or one out of every 15 people living in rural areas worldwide. From 1990–2010, the prevalence in low-middle income countries grew fourfold, while in high-income countries, the prevalence was twofold higher [3]. Furthermore, rural populations often live in worse socioeconomic conditions and have poor literacy skills compared with their urban counterparts [4]. As such, there is an urgent need to find innovative and effective solutions to help citizens in rural communities especially in low-middle income countries to successfully manage diabetes [5].

China has the largest number of individuals with diabetes mellitus; 113.9 million (11.6%) adults at age 18 or older have diabetes and 493.4 million adults (50.1%) are pre-diabetic. The prevalence of diabetes and pre-diabetes in rural areas is an estimated 10.3% and 50.9% respectively [6]. Considering the problem of a growing diabetic population, health professional shortages, and an under-financed health care system in China, a effective strategy is crucial. Providing education programs to a fast growing number of people with diabetes, particularly the poor and less-educated rural community, presents a huge challenge to Chinese health care system. To improve diabetes care and positively influence health outcomes, new approaches need to be developed that are both effective and feasible in Chinese rural communities [7].

Nowadays, peer support models have been widely recognized as a promising solution. The concept of peer support emphasizes a patient-centered approach as “the provision of emotional, appraisal and informational assistance by a created social network member who possesses experiential knowledge of a specific behavior or stressor and similar characteristics as the target population, to address a health-related issue of a potentially or actually stressed focal person” [8]. Although the WHO reviewed the use of peer support programs for people with diabetes and found numerous patient benefits, such as glycemic control and increase in quality of life, further evaluations are needed before peer support interventions can be integrated into the existing policy for diabetes management [9]. A systematic review by Jeremy Dale and colleagues found that peer support programs exhibit a latent potential towards improving health outcomes in adults living with diabetes. However the evidence-based review also identified the evaluation’s inconsistency and limitations, emphasizing the need for additional data related to cost effectiveness in future research [10]. This study will seek to show that peer support models provide a potentially low-cost, flexible compliment to formal health care services [11].Trained peer leaders can become extensions, or adjuncts to a formal healthcare system, thereby assisting with education delivery and bolstering the efforts of health care professionals [7]. In the current Chinese health care system, diabetes education has not been incorporated, and rural populations with diabetes have not yet acknowledged the responsibility for their own disease or adopted recommended health behaviors. A survey using anonymous standardized patients showed that baseline-educated rural clinicians are not yet prepared to act as the front line fighters in China’s primary care system, or equipped to tackle problems in health education [12].Therefore, exploring effective strategies to support diabetes in rural communities is desperately needed.

This article is a study protocol for a cluster randomised controlled trial for a peer support programs in a rural community within China. Our aim is to implement and evaluate a peer support program for people with type 2 diabetes within a specified local rural community. This program is designed to allow the participants to meet and share their self-management challenges, to explore and develop strategies to overcome these challenges, as well as sustain health behavior change using the experience and support derived from the groups. This study will give valuable information about the effectiveness, acceptability and feasibility of a novel way to improve diabetes self-management among developing countries.

## Methods

### Objectives

This study aims to implement and evaluate the biophysical and psychosocial outcomes of a culturally specific peer support program using a cluster randomized controlled trial in patients with type 2 diabetes and to determine whether it is an acceptable, cost effective intervention in the rural community health care centers. The study’s objectives are as follows:

1. To establish a culturally appropriate and feasible diabetes peer leader education curriculum by assessing participants’ adaptiveness to curriculum components, changes in trainees’ knowledge, attitudes, self-care skills and peer support outcomes.
2. To establish a diabetes management network between the hospital, the community, and peer groups, and explore its feasibility and sustainability.
3. To assess the following measures across peer support and control groups: HbA1c, AGE, blood lipids, blood glucose, blood pressure, BMI, medication adherence, knowledge, reported self-efficacy, self-care activities, quality of life, trait affect, life satisfaction, depression, anxiety, and stress.
4. To evaluate differences in cost efficiency between the intervention group and the comparison group.

### Study design and setting

This study is a cluster randomised controlled trial implemented and reported in accordance with the requirements of the CONSORT statement [13] and its extension to cluster randomised trials [14]. The participants were randomly designated to either an intervention arm, which implements the usual care system and the peer support intervention, or a control arm which implements a usual care system, with geographical location being the unit of cluster randomisation.

All participants are patients with type 2 diabetes who receive primary health care at 12 rural community health centres located in Liu-he District, Nanjing, and Gao-gou Town, Huaian, in Jiang Su province. The study team coordinates and works hand-in-hand with the staff members at each community health centres to implement and monitor the study.

**Figure 1:**
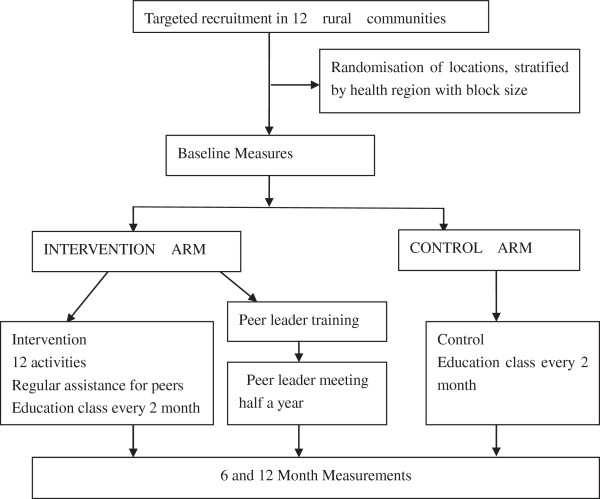
**Peer support for patients with type 2 diabetes in Nanjing--Consort diagram for diabetes project study design.**

Figure 1 is a CONSORT diagram of the study design.

### Ethics

Approval to conduct this study has been granted by the Committee on Human Research (Institutional Review Board) at Zhong Da Hospital, Southeast University (2012ZDLLKY05.0). Written informed consent was obtained from all trial participants after providing sufficient time to consider partaking in the study, read the details provided on the Patient Information Sheet, in addition to ask and have answered any questions pertaining to the trial.

### Eligibility criteria

#### Peer leaders

Inclusion criteria:

1. Patients with type 2 diabetes and HbA1c ≤ 7.5%;
2. Insulin injection experience is preferred,
3. Can commit to attending a 20-hour training, organize activities with other patients every month and attend a leadership meeting at least twice a year,
4. Have basic diabetes self-management knowledge and supportive, non-judgmental communication skills,
5. Willing to lead.

Exclusion criteria:

1. Patients with unstable mood or major psychiatric conditions,
2. Physical disability or severe speech impediment which may influence communication with others,
3. Patients with other serious health conditions (e.g., terminal cancer) or life expectancy less than 12 months,
4. Illiteracy.

#### Patients

Inclusion criteria:

1. Patients with type 2 diabetes,
2. Declaration of voluntary participation in the study with signed informed consent form,
3. Reside in local community during the intervention period.

Exclusion criteria:

1. Patients with unstable mood or major psychiatric conditions,
2. Patients with serious diabetes complications (e.g., blindness) that would impede meaningful participation in the program,
3. Patients with other serious health conditions (e.g., terminal cancer) or life expectancy less than 12 months,
4. Patients currently enrolled in other research program.

### Enrolment and randomization

#### Participants

Whether on self-recommendation or recommendation by clinicians and staff members, all peer leader candidates will follow a strict selection procedure from which eligible peer leaders are identified. Additional patients were also selected from primary health care settings and recruited from posted flyers. Primary care clinicians reviewed search results and excluded patients. The study team contacted eligible patients via phone calls to explain the study and arranged enrolment appointments.

#### Group allocation

Community health centres will be stratified by size and existing structure of primary diabetes care service. Allocation to intervention or control group is governed by a random number generation process using SAS 9.1 statistical software independently.

### Intervention

#### Peer leader training

Peer leaders are required to attend twenty-hour structured training programs in their community which were led by the study team. The training was conducted using a standardised training curriculum developed by the research team. The training team comprised of endocrinology physicians, certified diabetes educators (nurses), registered dieticians, exercise physiologists, psychiatrists, traditional Chinese medicine specialists, and communication trainers. The program aimed at enhancing diabetes knowledge and promoting diabetes self-care behavior. The Peer Leader Training (PLT) curriculum utilizes multiple educational approaches (such as lecture, practise, seminar and etc.) and consists of 5 major components. The content covers basic knowledge of diabetes, behavior change skills, and communication techniques, organisational management methods highlighting individual responsibilities and group management techniques, as well as psychological support.

#### Control group

The sessions for the control groups were interactive and informal. Participants were given a handbook that introduces issues in the training session. Patients attend the self-management training and communicate with the professionals every two months to obtain information on diabetes diet, exercise, glucose monitoring, etc. Additionally, group members will attend three follow ups at baseline, 6 months, and 12 months.

#### Intervention group

Patients are divided into groups of 8–15 people and assigned to peer leaders for 12 months according to their area of residence. In addition to receiving the same training and follow-ups as the control group, patients from intervention groups were suggested and encouraged to take part in monthly group activities with peer leaders. If possible, casual activities (such as phone calls, WeChat voice messages, physical exercises, group member’s family visits, shopping together, etc.) are also recommended. The first meeting is an introductory discussion of group members’s backgrounds and their disease history, through which the leaders established understanding and relationship with the peers. In subsequent interactions, the leader and peers will discuss the following issues: their current and target values for HbA1c, blood lipids and blood pressure; self management skills such as using a glucose meter and appropriate strategies for hypoglycaemia; lifestyle changes around healthy diets, physical activities, foot care, and stress control. The subsequent monthly activities include interactions designed by the research team, peer leaders, as well as college student volunteers. Several cultural group activities will be organized such as Chinese hand calisthenics and body calisthenics, how to choose daily nutritional meals, knowledge competition, and singing songs that were integrated with health education topics. Group members also shared similar experiences on lifestyle, family life, personal hobbies and experiences to support each other in achieving goals. Peer leaders documented each encounter and recorded each time the date, number of participants, the nature of encounter, approximate duration of contact, and topics discussed. In addition, peer leader meeting will be held every six months. In the leader meetings, peer leaders will share their experiences and discussed the problems they confronted in their groups. The research team will aid them discover the solutions and provide them with further training when necessary.

### Sample size calculation

Sample size and power calculations are performed for the main outcome of interest-difference in mean HbA1c levels-using effect sizes and standard deviations derived from multiple published trials of patient-education interventions. Using a non-inferiority design based on clinically relevant difference of HbA1c of 0.5%, standard deviation of 1.5, and groups of 8–15 participants per randomisation group for 80% power at two-sided 5% significance, 150 participants are required to complete the study procedures. Allowing for a 20% drop-out from randomisation to completion of the trial, it is anticipated that we would require 180 subjects in each arm to be randomised in the study.

### Data collection and outcome measures

Data will be collected at baseline, 6 months and 12 months for both intervention groups and comparison group. Table 1 shows the measurement tools of data collection, the method of data collection and the time points of data collection.

**Table 1:** **Measurement domains, survey tools used at each data collection time point**

Variable	Measurement tools/questions	Base-line	6mth	12mth
Demographic measures	Sex, age, ethnicity, religion, education, occupation, health insurance, marital status, anual income, annual household expenditure	√		
Health status	Time of diagnosis, family history, complications and co-morbidities, smoking, alcohol intake, frequency of physical exercise, length of diagnosis with diabetes, health education access;	√		
	therapy, list of a prescribed medications, events of hypoglycemia	√	√	√
Psychosocial status	Diabetes knowledge	√	√	√
	Activities of self-management, (SDSCA)	√	√	√
	Self-efficacy (Diabetes Self-Efficacy Scale)	√	√	√
	Adherence to medication (Morisky Scale)	√	√	√
	Quality of life( EQ5-D, DDS)	√	√	√
	Life satisfaction(SWLS)	√	√	√
	Trait affect(PANAS)	√	√	√
	Depression, anxiety and stress( PHQ-9, DASS)	√	√	√
Biomedical measures	Weight, , height	√	√	√
	Waist circumference, hip circumference	√	√	√
	Blood pressure,	√	√	√
	HbA1c(glycosylated haemoglobin)	√	√	√
	AGEs( advanced glycation end products )	√	√	√
	Blood glucose (fasting plasma glucose and 2 hour post load glucose)	√	√	√
	Blood Lipids(total cholesterol, low density lipoprotein, high density lipoprotein, triglycerides)	√	√	√
Cost	Cost of medicine, travel time and fee, escort fee, hospital stays and fee, cost of services, etc.	√	√	√

Data collection consists of two main parts, questionnaire and biophysical data. The questionnaire data includes four main parts and will be collected by trained investigators. The first part covers social demographics (e.g., sex, age, ethnicity, religion, education level, occupation, health insurance, marital status, annual income, etc.); the second portion concerns measures of health status (eg, time of diagnosis, smoking, alcohol intake, frequency of physical exercise, therapy, length of diagnosis with diabetes, family history, complications and co-morbidities, educational history and type, glucose monitor). The third section is psychosocial status including knowledge of diabetes; activities of self-management [15], self-efficacy [16], adherence to medication [17, 18], quality of life [19–21], diabetes distress screening [22, 23], traits [24], life satisfaction [25], depression, anxiety and stress [26–28]. The final section asks about economic status, which covers training, equipment availability, human resources, drugs, travel fees, escort fees, communication expenses, consumables, etc.

The biophysical data are collected by specifically trained staff according to standard operation procedure. Height is measured using a portable height rod to the nearest 0.1 cm with the participants standing without headgear or shoes. Weight is measured to the nearest 0.1 kg using a calibrated, portable, digital weighing scale. Body mass in dex (BMI) is calculated as weight in kilograms divided by height in meters squared. Waist circumference is measured midway between the lowest rib and the top of the hip bone using the tape measure. Hip circumference is taken at the maximum circumference over the buttocks [29]. Duplicate waist and hip circumference measurement is taken for approximately 10% of the participants for quality assurance. Blood pressure meter and weight scales are calibrated at least weekly. Laboratories providing blood analysis are accredited, and all methods for determining HbA1c% are NGSP approved.

### Outcome measures

Outcomes variables are measured at baseline, 6 months, and 12 months, to both participants and peer leaders. The primary expected outcome is the change in HbA1c at baseline compared to the sixth month and baseline to the twelfth month. HbA1c, a measurement of glycosylated haemoglobin, is regarded as the gold standard measure of glycemic control reflecting overall blood glucose values over the previous 8–12 weeks [30, 31]. There is a strong relationship between HbA1c and the risk of developing long-term diabetic complications and it is accepted as a reasonable surrogate for long-term outcomes in individuals with diabetes. Secondary outcomes are advanced glycation end products (AGEs) [32, 33], blood pressure, BMI, blood lipids, blood glucose (fasting plasma glucose and 2 hour post load glucose), patient-reported rates of severe hypoglycaemia, diabetes self-care activities, medication adherence, knowledge, quality of life, diabetes self-efficacy, traits, life satisfaction, depression, anxiety, and stress. In addition, cost is also assessed.

### Data analysis

Social demographic data and health status of the study subjects are presented as frequencies (percentage) for categorical variables, and means ± standard deviations for continuous variables. Comparison of patients characteristics between groups will be checked by means of T-tests or χ^2^ tests, or non-parametric equivalents. Repeated measures analysis of variance and multilevel analysis for longitudinal data will be used to examine longitudinal differences between groups on primary and secondary outcome measures. Evaluation of intervention effectiveness will be by intention to treat using the above statistical tests. Evidence of clustering by clinic site and primary care provider will be examined and adjusted for analyses as needed. If significant differences in baseline characteristics are found, analyses will be repeated adjusting for these differences using ANOVA and logistic regression for multivariate analyses. Analysis of covariance (i.e. linear regression models for 12-month measurements with baseline scores and intervention arm as covariates) will estimate the changes from baseline to 12 months that can be attributed to the peer support intervention. Sensitivity analyses will be performed to estimate the effects of missing data using different assumptions (e.g. imputed values).The EuroQoL 5 dimensional health state measure (EQ5D) score at base-line and follow-up can be converted into utility weights using the EQ5D algorithm [34], which then allow for the calculation of quality adjusted life years(QALYs). Economic analyses will be employed to estimate direct and indirect cost analysis, the cost per QALY gained and incremental cost-effectiveness ratios. Sensitivity analysis will be undertaken to test the stability of the analysis in terms of the cost inputs and health outcomes. All analyses will be carried out with SPSS 10.0 software.

### Qualitative evaluation

A descriptive parallel qualitative analysis is being carried out to record the attitude of patients, professionals, educators, rural clinicians and peer leaders to peer support and their experience of delivery in intervention practices. The peer leaders will voluntarily participate in a focus group, semi-structured interviews, or both, to assess how they experience the training and leading process. The focus group is designed to reveal the peer leaders’ general attitude and facilitate the development of the interview guide. Peer leaders are encouraged to discuss their feelings and efficacy, the training experience, motivations, as well as any positive and negative impacts on their own diabetes management. Semi-structured qualitative interviews and focus group with diabetic patients will also be carried out and analysed through the investigation and daily observations. The focus group and interviews are audio recorded and transcribed.

## Discussion

The World Health Organization’s action plan for chronic disease management helps encouraged governments to take action to aid people to better managing their own chronic conditions by providing education, incentives, self-management tools and care [35]. Implementation of a diabetes management plan should include clinical care, diabetes self-management education and ongoing support [36]. Self-management training and support is an effective component of care for people with diabetes. Due to a shortage of time and resources, this critical support is not consistently delivered in most health care settings. The study uses trained patients to provide peer support in urban community. We anticipate that patients in the intervention arm with a peer leader will show significant improvement in the biophysical and psychosocial outcomes, and so will the peer leaders. In addition, we hypothesize that the recruitment and training of volunteers with type 2 diabetes as peer leaders to support participants with the same conditions will be a cost-effective strategy. We expect that the qualitative and health economic research will reveal an in-depth perspective on the impact of this approach both at the level of the patient as an individual and at the societal level. Successful implementation of this trial will provide evidence of the key functions of peer support, identified by the global Peers for Progress program. Moreover, the results of this study will influence future policies on diabetes management, as well as other chronic diseases in China.
